# Protein feature engineering framework for AMPylation site prediction

**DOI:** 10.1038/s41598-024-58450-8

**Published:** 2024-04-15

**Authors:** Hardik Prabhu, Hrushikesh Bhosale, Aamod Sane, Renu Dhadwal, Vigneshwar Ramakrishnan, Jayaraman Valadi

**Affiliations:** 1 Computing and Data Sciences, FLAME University, Pune, 412115 India; 2 Bioinformatics Center, School of Chemical and Biotechnology, SASTRA Deemed to be University, Thanjavur, 613401 India; 3 Present Address: Robert Bosch Centre for Cyber Physical Systems, Indian Institute of Science, Bengaluru, 560012 India

**Keywords:** Computational biology and bioinformatics, Mathematics and computing

## Abstract

AMPylation is a biologically significant yet understudied post-translational modification where an adenosine monophosphate (AMP) group is added to Tyrosine and Threonine residues primarily. While recent work has illuminated the prevalence and functional impacts of AMPylation, experimental identification of AMPylation sites remains challenging. Computational prediction techniques provide a faster alternative approach. The predictive performance of machine learning models is highly dependent on the features used to represent the raw amino acid sequences. In this work, we introduce a novel feature extraction pipeline to encode the key properties relevant to AMPylation site prediction. We utilize a recently published dataset of curated AMPylation sites to develop our feature generation framework. We demonstrate the utility of our extracted features by training various machine learning classifiers, on various numerical representations of the raw sequences extracted with the help of our framework. Tenfold cross-validation is used to evaluate the model’s capability to distinguish between AMPylated and non-AMPylated sites. The top-performing set of features extracted achieved MCC score of 0.58, Accuracy of 0.8, AUC-ROC of 0.85 and F1 score of 0.73. Further, we elucidate the behaviour of the model on the set of features consisting of monogram and bigram counts for various representations using SHapley Additive exPlanations.

## Introduction

When it comes to predicting AMPylation sites, the effectiveness of machine learning models depends significantly on the selection of features used to represent the raw protein sequences. In our research, we introduce a feature extraction framework aimed at encoding the essential properties relevant to AMPylation site prediction. We then select a black-box machine-learning model from a variety of options for this task, which includes options from Tree-based Ensembles to Artificial Neural Networks. Additionally, we evaluate the performance of the models with various extracted features using our framework and select the top-performing ones. Subsequently, we elucidate the black-box model behaviour on the best set of extracted features using our method through the application of SHAP (SHapley Additive exPlanations).

The subsequent sections of this paper are organized as follows: We provide a review of related work, which includes studies on AMPylation site prediction and various feature engineering techniques. We also touch upon the subject of explainability. An overview of the dataset employed in our experiments is presented, followed by a deep dive into our feature engineering framework. We then provide a comprehensive description of our end-to-end modelling, and an exploration of the SHAP method for model explainability. The outcomes of our experiments are detailed, and we conclude with our final remarks.

## Related works

### Post translational modifications

Post translational modifications (PTMs) are covalent modifications of a protein that involve proteolytic cleavage or modification of one or more amino acid in the protein with functional groups such as acetyl, phosphoryl, glycosyl, methyl, etc.^1^. Such modifications play an important role in the functioning of the proteins, and the biological processes they are involved in. A noted example of a protein undergoing post-translational modification is the tau protein which undergoes a variety of modifications such as phosphorylation, glycation, ubiquitination etc^2,3^. Tau protein is implicated in the Alzheimer’s Disease. The acetylation of the transcription factor Twist is implicated in breast cancer metastasis^4^. Palmitoylation and glycosylation of the spike protein of the SARS-CoV-2 virus has been shown to be important for its host cell receptor interaction^5^. Thus, post translational modifications play an important role in protein function and is involved in the progression of several diseases. Consequently, the enzymes involved in modifying the proteins have been promising therapeutic targets. Tyrosine kinase inhibitors which prevent the phosphorylation of tyrosine residues is a classic example of the same^6^. Thus, identification of post translational modifications can shed light into disease pathogenesis and can form the basis for therapeutic interventions. Experimental identification of post translational modification is usually done using mass spectrometry. However, this approach is time-consuming, labor-intensive and expensive^7^. Therefore, there has been a great interest in developing computational methods for predicting post translational modification sites in protein sequences. Zhao et al.^8^ developed an iterative semi supervised learning technique to identify succinylation sites in proteins. Saethang et al.^9^ developed a machine learning strategy for predicting post translational modification sites in protein-protein interaction regions. Chandra et al.^10^ used structural properties of amino acids to predict phosphoglycerated lysine residues. Chung et al.^7^ used physiochemical properties, evolutionary information and sequence-based features for identifying malonylation sites in mammalian proteins. Naseer et al.^11^ developed a deep learning model to predict 4-carboxyglutamate sites in proteins. Liu et al.^12^ developed a computational method for predicting lysine glycation sites for *Homo sapiens*. Alkuhlani et al.^13^ used protein language models and deep learning methods to predict post-translational glycosylation and glycation sites. Thus, we see that there have been several computational methods for predicting different post-translational modification sites in protein sequences.

### AMPylation site prediction

AMPylation is a PTM that is gaining attention^14–16^. AMPylation is mediated by bacterial virulence factor containing the ‘Fic’ domain that transfers AMP to the threonine or the tyrosine residues in the eukaryotic substrates^14^. It has been shown that the addition of AMP to Rho-family GTPases mediates bacterial pathogenesis and as well eukaryotic signaling^17^. Truttmann et al. show that AMPylation of chaperones modulates aggregation and toxicity of polypeptides involved in neurodegenerative diseases^18^. In general, AMPylation has been implicated in neurodevelopment and neurodegenerative diseases^19^. Recently, it has also been shown that AMPylation can act as a molecular rheostat tempering the unfolded protein response during physiological stress in the pancreas^16^. Consequently, identifying whether a protein undergoes AMPylation assumes biological importance. Azim et al.^20^ develop a convolutional network-based tool for predicting AMPylation sites. Their method achieves an accuracy of about 78% with an MCC of 0.55. However, their approach involves straightforward feature engineering based on a binary profile. In our research, we place a strong emphasis on feature extraction, as we believe it significantly contributes to the accuracy of the model. In our work, we present a feature extraction framework which combines a wide range of feature extraction methodologies. In the next subsection, we give a brief review of the existing work done on feature extraction from raw protein sequences.

### Feature extraction

Amino acid composition has been used as a feature to predict the functional properties of proteins. For instance, King et al.^21^ use amino acid composition-based features for predicting the functional class of proteins in the *Mycobacterium tuberculosis* and *Escherichia coli*. Jensen and Brunak^22^ use amino acid composition features, in addition to other features, to predict novel archaeal enzymes. Liu et al.^23^ use pseudo amino acid composition to predict DNA binding proteins. Further, dipeptide compositions have also been used as features in machine learning problems in protein functional annotation. These include ungapped or gapped dipeptide compositions. Gapped dipeptide compositions serve to capture the higher-order residue relationships in a protein sequence and have been successfully used in the prediction of cancerlectins^24^ and several post-translational modification sites such as N-glycosylation^25^, phosphoglycerylation^26^ and succinylation^27^.

Reduced alphabets based on domain information and similarity measures have been used by several researchers successfully for various prediction problems. These include the identification and classification of GPCRs^28^, prediction of DNA-binding proteins^29^, protein folding^30^ etc. Distributed representations of words and phrases in textual data has recently gained much attention owing to its potential to capture semantic relationships. The methodology, pioneered by Mikolov and coworkers^31–33^, was used for biological sequence representation in protein family classification and classification of disordered proteins^34^. Subsequently, various researchers have applied variants of distributed representation of biological sequences in various classification problems including splice junction prediction^35^, phosphorylation site prediction^36^, predicting lncRNA-protein interactions^37^ etc. Wijesekara et al.^38^ combined the use of distributed representation and reduced alphabet representation of protein sequences. This method, known as RA2Vec has been shown to be successful in predicting phase-separating proteins and pore-forming proteins.^39,40^. Additionally, several outstanding protein language models have been utilized for the identification of post-translational modification sites.^41–43^.

Gray level co-occurrence matrix (GLCM) is a popular second-order statistical method that has been widely used for texture analysis in bioinformatics applications^44^, especially for histopathology^45^ and microscopy image analysis^46^. GLCM captures the spatial co-occurrence patterns of gray levels, enabling quantification of texture properties like homogeneity, contrast and entropy to represent architectural patterns in biological images. Studies have utilized GLCM features with machine learning for various bio-image classification tasks. Additionally, beyond images, this methodology extends to gene nucleotide sequences, where co-occurrence matrices illustrate the statistical distribution of stationary nucleotide patterns. These matrices yield texture features like energy, entropy, and contrast^47^. Texture features computed from these matrices can then be used with machine learning for essential gene classification.

### Model explainablility

The significance of explainability within the AI and ML domain has grown substantially as more intricate models have emerged. These models, often referred to as black box models, tend to lack transparency and offer limited insights into their internal operations. Acknowledging this, Burkhart and Huber^48^ carried out a comprehensive examination that delves into the principles and methodologies governing explainable Supervised Machine Learning.

In a separate review conducted by Vilone and Longo^49^, various theories related to explainability and evaluation methods for Explainable AI (XAI) techniques are categorized and explored. This review also critically assesses the existing gaps and constraints in this field while suggesting potential avenues for future research.

Explainability methods can be broadly classified into two categories: intrinsic and post-hoc approaches. Intrinsic methods, also known as transparent or interpretable models, are models that inherently possess interpretability and feature easily understandable decision-making mechanisms. Instances of intrinsic models encompass Decision Trees, Linear Regression, and Logistic Regression. These models naturally offer insights into the decision-making process.

Conversely, post-hoc methods entail the interpretation of predictions made by black-box models without altering the models themselves. An example of such a method is SHAP^50^ (SHapley Additive exPlanations), which can be applied to any black-box model without being reliant on the specific architecture or design of the model. In our study, we will utilize the SHAP method to provide explainability for the black-box model we have employed.

## Dataset

The dataset is the same as that used by Azim et al.^20^. Briefly, this includes the proteins identified to undergo AMPylation in intact cancer cells such as the HeLa, SY-5Y etc^51^. LC-MS/MS and imaging methods were used to identify the sites of protein modification. A total of 162 proteins involved in various metabolic pathways were found to have undergone AMPylation. These were mapped to 133 unique protein sequences in the Uniprot database. After removal of redundant sequences at 40% similarity level, the resulting positive dataset constituted 130 protein sequences with 153 AMPylated sites. For each of the AMPylated and non-AMPylated site, a 31-residue peptide was extracted by using 15 amino acids upstream and downstream of the site. The procedure resulted in a total of 403 peptide sequences with 153 AMPylated and 250 non-AMPylated sites. In their work AMPylated sites were identified using N6-propargyl adenosine phosporamidate proneucleotide probe. Based on this, they had identified the AMPylated and non-AMPylated sites. To keep a constant length of 31 peptides Azim et al.^20^ had used padding with a random “X” residue in 27 sequences. In our work we removed these sequences. Our final data set consists of 378 protein sequences, which we have made available online.

## Feature extraction methodology

### Protein sequence and reduced alphabet representation

Proteins are made up of amino acids that are linked together in a long chain. The composition of these amino acids determines its function. A single change in the amino acid sequence can lead to significant alterations in protein structure and, consequently, its role in the cell. Understanding the protein sequence is vital for comprehending how genetic information is translated into functional molecules that carry out essential tasks in living organisms. There are 20 common amino acids that can be incorporated into proteins. Each amino acid is represented by a 1 letter abbreviation. A protein sequence uses these amino acid abbreviations as characters to indicate the order of amino acids in the protein.

There are several ways to reduce the total number of unique alphabets in a given sequence by grouping amino acids together based on certain properties. Hence providing alternate representations of a given sequence. In addition to the standard representation (20 unique alphabets), we also consider reduced representations based on conformational similarity index (7 unique alphabets) and hydrophobicity (5 unique alphabets).

1. Based on conformational similarity index: Amino acids grouped based on similarity of phi/psi angle shifts.
2. Based on hydrophobicity: Amino acids grouped based on normalized Kyte-Doolittle hydropathy score ranges.

**Figure 1:**
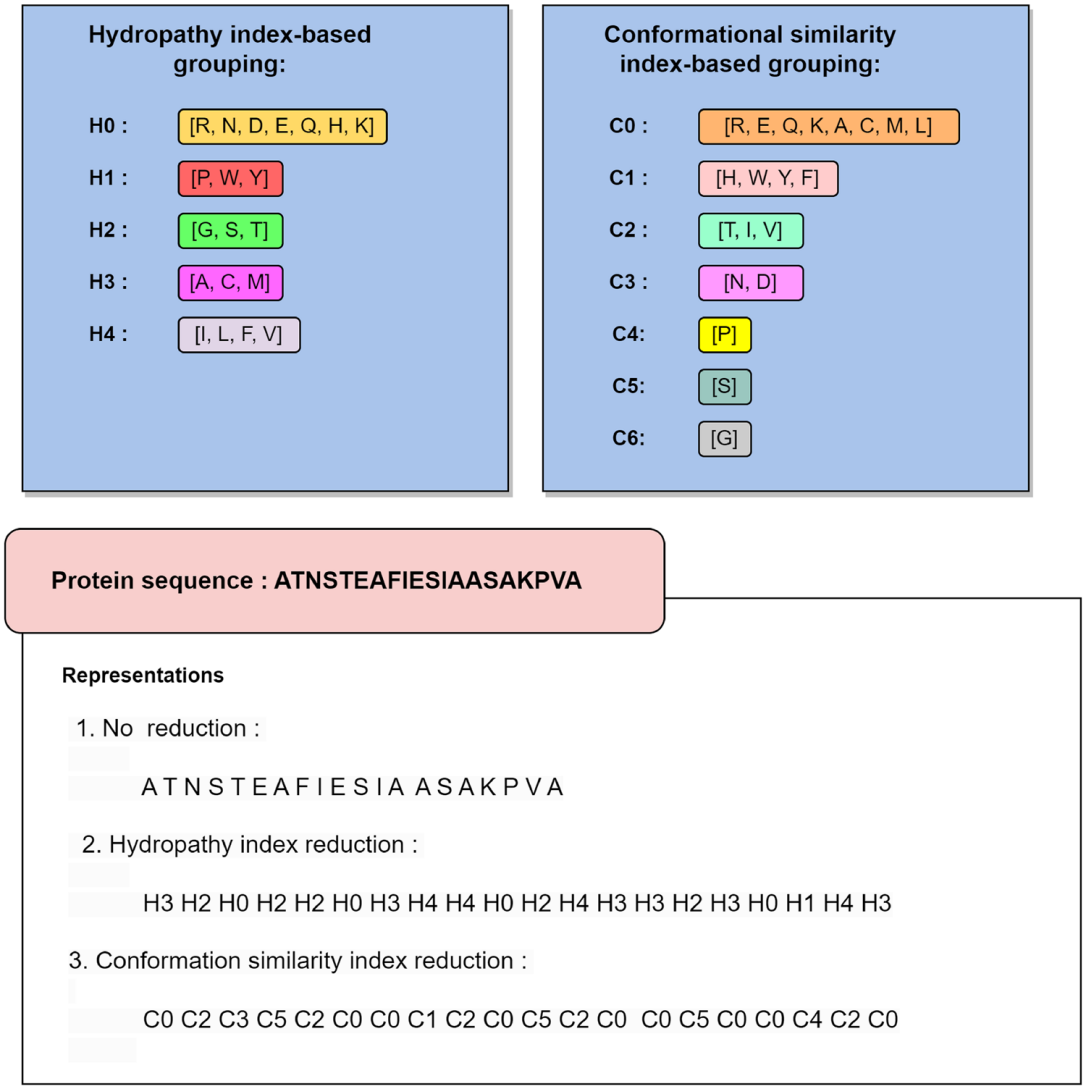
Protein sequence representation schemes.

The conformational similarity-based reduction was earlier used by Idicula-Thomas et al.^52^ based on the reduction scheme proposed by Pal and Chakrabarti^53,54^. It has been subsequently used in the prediction of phase-separating proteins^39^ and pore-forming proteins^40^ as well. The conformational similarity index groups the amino acids based on the interrelationship of the side-chain and main-chain conformations in the protein. In essence, this reduction in the alphabet is based on the conformational similarity of the amino acids. The Kyte-Doolittle hydropathy index is calculated based on the water-vapour transfer free energies and the residue accessibility determined by the interior-exterior distribution of amino acids^55^. It has been successfully used in several classification problems including in discriminating soluble proteins from membrane proteins^56^, disorder region prediction^57^ etc. Figure 1 provides an example of how a protein alphabet sequence could be converted into either of the two reduced alphabet representations.

### Counts and co-occurences

In order to obtain numerical attributes from the sequences of alphabets, researchers frequently establish a frequency-oriented portrayal of individual characters (monograms) and pairs of contiguous characters (bigrams), often termed monopeptide and di-peptide counts when addressing amino acids in the context of protein sequences. As we are not solely focused on the primary character sequence of proteins but also consider a sequence of simplified characters, we will persist with the monogram and bigram terminology. This feature extraction process involves counting the occurrences of each alphabet and pairs of adjacent alphabets in the sequence. By doing so, a numerical representation is obtained for the alphabet sequence, reflecting the composition and local order.

#### Monogram counts

Let *S* be an alphabet sequence of length *n*, represented as:

1 $$\begin{aligned} S = [a_1, a_2, \ldots , a_n] \end{aligned}$$

The monogram counts feature vector, $C_{\text {mono}}$, of length k (k representing the unique characters) is defined as follows:

2 $$\begin{aligned} C_{\text {mono}}[j] = \text {Count}(p_j) \quad \text {for }\, j = 1 \,\text { to } \,k \end{aligned}$$

Where $\text {Count}(p_j)$ is the function that returns the number of occurrences of the alphabet $p_j$ in the sequence *S*. The count vector is then normalized. Let $S_{\text {total}}$ represent the sum of counts of all elements in the feature vector $C_{\text {mono}}$:

3 $$\begin{aligned} S_{\text {total}} = \sum _{j=1}^{k} C_{\text {mono}}[j] \end{aligned}$$

For each element $C_{\text {mono}}[j]$ of the feature vector $C_{\text {mono}}$, the normalized vector is calculated as follows:

4 $$\begin{aligned} C_{\text {mono}}[j] = \frac{C_{\text {mono}}[j]}{S_{\text {total}}} \quad \text {for }\, j = 1 \text { to } \,k \end{aligned}$$

#### Co-occurance matrix and bigram counts

Bigram counts can reveal patterns in the local structure of a protein. Certain amino acid pairs may have a higher propensity to form specific secondary structures like alpha-helices or beta-sheets. By analyzing bigram frequencies, researchers can gain insights into the likely folding patterns of the protein. To achieve this, researchers commonly employ co-occurrence matrices, which effectively capture the distribution of the local patterns of amino acids in the protein sequence. These co-occurrence matrices provide valuable information about the local relationships and interactions between amino acids, shedding light on the structural and functional characteristics of the protein.

To construct a co-occurrence matrix for a given alphabet sequence, bigram counts are utilized, resulting in a matrix denoted as *G*, with dimensions $k \times k$. Each element $G_{ij}$ in this matrix corresponds to the count of the bigram formed by alphabets $p_i$ and $p_j$ within the alphabet sequence. The calculation for $G_{ij}$ is defined as follows:

5 $$\begin{aligned} G_{ij} = \text {Count}(p_i, p_j) \quad \text {for } \,i = 1 \,\text { to }\, k, \text { and } j = 1\, \text { to }\, k \end{aligned}$$

where ${\text {Count}}(p_i, p_j)$ represents a function that returns the number of occurrences of the bigram “$p_ip_j$” in the alphabet sequence *S*. The co-occurrence matrix, based on bigram counts, provides valuable information about the local patterns of amino acid pairs within the protein, which is instrumental in understanding its structural and functional attributes.

Bigrams with offsets: Counting bigrams with offsets is a method used to analyze the co-occurrences of alphabets at specific relative positions within a sequence. The process involves systematically scanning through the alphabet sequence and identifying pairs of alphabets separated by a fixed offset ($\delta$).

To perform the counting, we start by iterating through the sequence, considering each alphabet as the reference point (denoted as $a_t$). We then look for neighbouring alphabet at positions $t+\delta$ within the sequence, where $\delta$ is the fixed offset.

For each pair of alphabets $a_t$ and $a_{t+\delta }$ with the given offset, we increment the count of the corresponding bigram formed by these two alphabets. This count is stored in a bigram co-occurrence matrix $G^{\delta }$, where each element $G^{\delta }_{ij}$ corresponds to the count of the bigram formed by alphabets$p_i$ and $p_j$ with the offset $\delta$ in the protein sequence.

6 $$\begin{aligned} G^{\delta }_{ij} = \text {Count}\,(a_t = p_i, a_{t+\delta } = p_j) \quad \text {for } i = 1 \text { to }\, k, \text { and } j = 1 \text { to }\, k \end{aligned}$$

By systematically analyzing the occurrences of bigrams with different offsets, we can obtain multiple bigram count matrices $G^{\delta _1}$, $G^{\delta _2}$, and so on, each providing valuable information about the spatial relationships and interactions of specific pairs of amino acids within the protein. Note that $\delta =1$ corresponds to the co-occurrence matrix *G*.

Finally, the co-occurrence matrix is normalized. Let $\text {Sum}$ represent the sum of all elements in the co-occurrence matrix $G^\delta$:

7 $$\begin{aligned} \text {Sum} = \sum _{i=1}^{k}\sum _{j=1}^{k} G^\delta _{ij} \end{aligned}$$

For each element $G^\delta _{ij}$ in the co-occurrence matrix $G^\delta$, the normalized value $G^\delta _{ij}$ is computed as follows:

8 $$\begin{aligned} G^\delta _{ij} = \frac{G^\delta _{ij}}{\text {Sum}} \quad \text {for } \,i = 1\, \text { to }\, k\, \text { and }\, j = 1\, \text { to }\, k \end{aligned}$$

The normalized $\delta$-bigram counts could be used directly as a feature vector after flattening $G^\delta$.

9 $$\begin{aligned} C_{\delta \text {-bigram}} = [G^\delta _{11}, G^\delta _{12}, G^\delta _{13},\ldots G^\delta _{ij}\ldots G^\delta _{kk}] \end{aligned}$$

### Deriving texture features

Additionally, to further characterize the protein sequences and facilitate discrimination, various discriminant features could be computed from each co-occurrence matrix^47^. The computed features include energy, entropy, homogeneity, contrast, and dissimilarity. The mathematical formulae are described below. Discriminant features are properties or characteristics of the data that can help differentiate or classify different samples.

1. Energy: It is a measure of the overall intensity or strength of the relationships between pairs of elements in the co-occurrence matrix. This could help reveal the overall significance or prominence of certain interactions within the protein sequence.

10 $$\begin{aligned} \text {Energy} = \sum _{i=1}^{k}\sum _{j=1}^{k} {G^\delta _{ij}}^2 \end{aligned}$$

2. Entropy: It is a measure of the randomness or uncertainty in the distribution of interactions. This could provide insights into the diversity of interactions and potentially highlight regions of interest where interactions are particularly complex or varied.

11 $$\begin{aligned} \text {Entropy} = - \sum _{i=1}^{k}\sum _{j=1}^{k} G^\delta _{ij} \log _2(G^\delta _{ij} +\varepsilon ) \end{aligned}$$

3. Homogeneity: It measures the similarity or uniformity of interactions within the co-occurrence matrix. It could indicate areas of consistency or regularity in the sequence’s interactions.

12 $$\begin{aligned} \text {Homogeneity} = \sum _{i=1}^{k}\sum _{j=1}^{k} \frac{G^\delta _{ij}}{1 + (i - j)^2} \end{aligned}$$

4. Contrast: It measures the variation in interactions between pairs of elements. This could help identify regions where interactions change abruptly, which might correspond to functional boundaries or transitions.

13 $$\begin{aligned} \text {Contrast} = \sum _{i=1}^{k}\sum _{j=1}^{k} (i - j)^2 \cdot G^\delta _{ij} \end{aligned}$$

5. Dissimilarity: It quantifies the differences between interactions. It could be used to highlight areas of the sequence where interactions are distinct, potentially indicating unique features or regions.

14 $$\begin{aligned} \text {Dissimilarity} = \sum _{i=1}^{k}\sum _{j=1}^{k} |i - j| \cdot G^\delta _{ij} \end{aligned}$$

### Pro2vec embeddings

#### Word2Vec

Word embeddings are numerical representations of a given word and is useful in Natural language processing and text mining tasks. These embeddings, commonly used for performance enhancement, can be mapped into different vector representations using various methods. Word2Vec, is a very popular method first proposed by Mikolov and coworkers which is employed to learn vector embeddings^31–33^. CBOW and Skip-Gram are two different basic word2Vec formulations. Employing a given word in a corpus the Skip-Gram model predicts its neighbors (context words). The CBOW model uses the converse, i.e., starts with the surrounding contexts to predict the current word. The surrounding words can be controlled by specifying appropriate window size parameters. In our work we have employed the Skip-Gram model along with the standard neural network architecture consisting of a single hidden layer. Taking every word in the corpus as input the model learns the optimal set of weights which maximize prediction of context words. The learnt weight vectors are the distributed representations. These vector representations of a fixed size are the embedding vectors which efficiently capture the semantic information. Thus, for every word in the original corpus we obtain a vector representation of a certain fixed size. This learnt vector representation is further employed as attributes in machine learning tasks. This distributed representation of words has been found to be a very efficient way to represent semantic information.

#### ProtVec

The Word2Vec model was first applied to handle biological sequences by Asghari and Mofrad^34^. In this novel embedding methodology, named ProtVec, the n-grams derived from the protein sequence, by converting the sequence of k characters into the k sets of shifted overlapping n-grams, are considered as words, and the protein sequence is considered as a sentence in a text corpus. The Skip-Gram algorithm extracts the vector representations of the word. The vector representation of the sentence (sequence) vector is the sum of the vectors of words it contains. Since the first-ever introduction of this novel distributed representation of biological sequence, several authors have demonstrated the usefulness of the approach in many case studies and have developed other different novel representations following the same approach in the biological context^58–61^.

#### RA2Vec

A novel method employing the distributed representation of protein sequence and combining it with reduced alphabets was proposed by Wijesekara et al.^38^. In this method, a protein sequence is first converted into a reduced alphabet sequence (based on the hydropathy index and conformational similarity index). The reduced alphabet version of the sequence is then converted to sets of shifted non-overlapping n-grams (words). The word embeddings are then learned using the skip-gram algorithm. In the context of our study, we define any process that transforms a protein sequence into a numerical vector-whether using the original sequence (as in protvec) or a reduced alphabet sequence (as in RA2Vec) under the collective term “Pro2vec.” For every given sequence all overlapping n-gram words are extracted by a pre-trained language model and summed up to get the embedding vector for that sequence. Thus the the vector size of each sequence will be equivalent to the embedding vector size of the n-gram. These embedding vectors are used as input to the classifier for building different models. In our studies, the dimensionality of the ProtVec and RA2Vec embedding vectors are 50 and 300, respectively.

### Feature extraction framework

**Figure 2:**
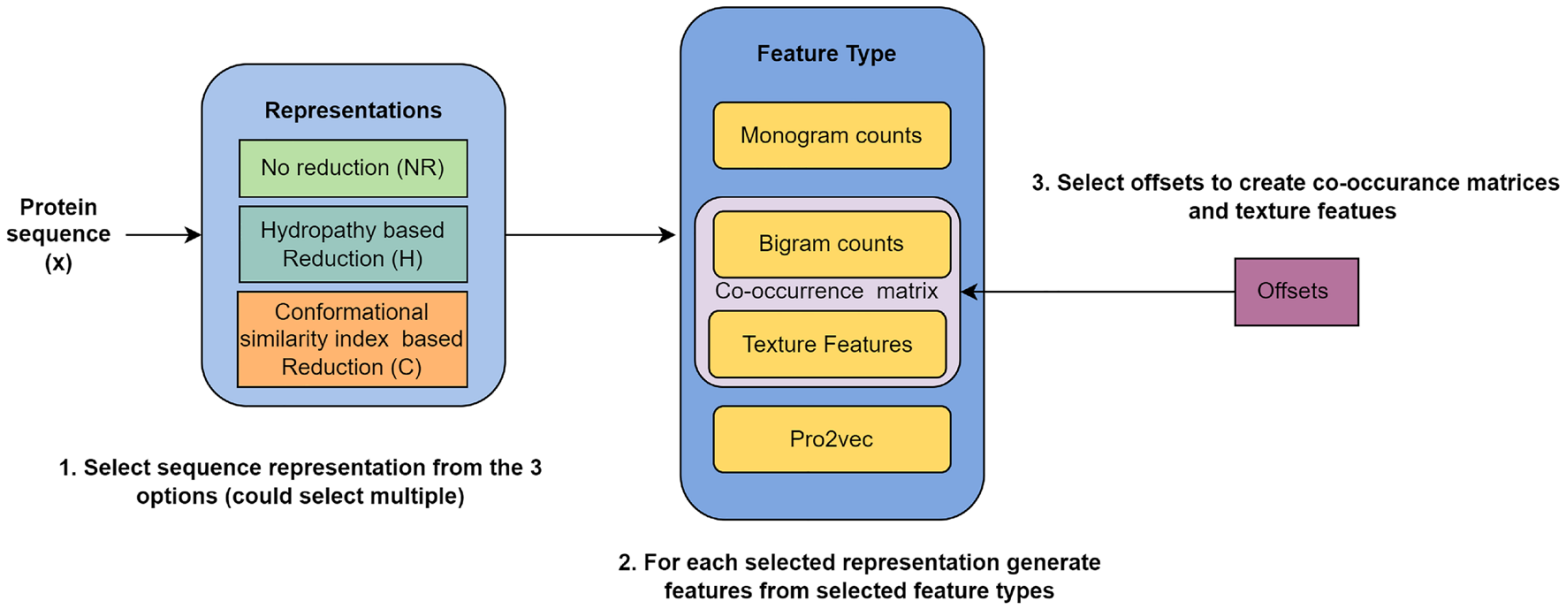
Feature extraction framework.

Our feature extraction framework operates on an input protein alphabet sequence *S* and constructs a feature vector for the given sequence. This process involves utilizing several lists as parameters: i. Representations, ii. Feature types, and iii. Offsets. The illustration of this feature extraction framework is depicted in Fig. 2. Below, we provide a detailed explanation of the process.

1. First, we select a subset from the 3 sequence representations (“NR”: No Reduction, “H”: Hydropathy Reduction, “C”: Conformational Reduction). For example, if we select the subset [“NR”, “H”], we have two alphabet sequences $S_1 = S$ (no reduction) and $S_2$ (hydropathy reduction). These sequences are then processed independently.
2. Next we select the type of features to be generated, the options are monogram counts, $\delta$-bigram counts (derived from the co-occurrence matrix), texture features (derived from the co-occurrence matrix) and pro2vec embeddings.
  For example, if we select [mono counts, texture features], sequences $S_1$ and $S_2$ would be separately processed to obtain, the count vectors of sizes 20 (for $S_1$) and 5 (for $S_2$) respectively, and 5 texture features each derived from the co-occurrence matrices.
3. We also accept a list of offsets to consider to extract the various co-occurrence matrices ($G^\delta$). Note that feature types, pro2vec and monogram counts are not affected by the offsets, only the texture features and bigram counts derived from $G^\delta$ depend on the offset value. For example, if we select offsets as [1,3], four co-occurrence matrices are created separately for the two offsets (two per two the sequences), $\delta =1$ and $\delta =3$, and then texture features are created (5 per matrix).
4. All the different features are then concatenated to create a vector. For the selected parameters of the framework (representations: [“NR”, “H”], feature types: [mono counts, texture features], offsets: [1,3]), we get: 5 + 20 (monogram counts), and 20 (texture features) to get a total of 45 features.

## Data driven AMPylation site classification

We apply our proposed feature extraction framework for an important post-translational modification classification task—identifying AMPylation sites in proteins. Given a protein sequence, the task is to classify if it is an AMPylation site or not. We extract numerical features as per our framework from alphabet sequences. For the subsequent prediction task, a machine learning model is chosen from a set of options, and this model is applied across different combinations of features derived from our framework.

### Logistic Regression (baseline model)

As our baseline, we select a Logistic Regression. It is the only fully transparent model among all our model options. Logistic Regression models the probability that a given input belongs to the positive class through a linear combination of the input features, providing a straightforward method for binary classification. By setting a threshold for the probability value, two classes could be separated by a linear hyper-plane.

### Support Vector Machines

Unlike Logistic Regression, which relies on linear decision boundaries to separate classes, Support Vector Machines (SVM)^62^ enable non-linear separation by constructing a linear hyperplane in a transformed high-dimensional space. This is achieved through the maximization of the margin between the two classes, where the margin represents the distance between the nearest points of the classes to the hyperplane, known as support vectors. SVM creates separating hyperplanes in higher dimensions without the need for explicitly mapping data to higher dimensions by application of the kernel trick. To calculate probabilities in SVM, a method known as Platt scaling is commonly used. Platt scaling involves fitting a logistic regression model to the scores (distances to the decision boundary) produced by the SVM.

### Artificial Neural Networks

Artificial Neural Networks (ANNs)^63^ work by sequentially performing non-linear transformations of the data one after the other by passing through layers. The layers are made of small computational units known as neurons which are nothing but linear transformations followed by non-linear activation functions.

Artificial Neural Networks (ANNs) perform a series of non-linear data transformations by employing a structured network of layers that sequentially process the input data. These layers are composed of fundamental units called neurons, each of which performs a linear transformation followed by a non-linear activation. The binary classification task can be viewed as non-linearly transforming the data by the application of hidden layers and then performing a logistic regression at the end by employing a neuron with sigmoid activation.

The model architecture adopted for the study is simple, consisting of three fully connected layers. It begins with a layer matching the input’s dimensionality, followed by a dimension-reducing layer capped at either 10 or half the input size, and concludes with a single-output layer applying a sigmoid function for probability estimation.

### Ensemble of trees—Random Forest, XGBoost and LightGBM

- **Random Forest:** Random Forest (RF)^64^ is a popular ensemble classification procedure based on bagging, which employs bootstrap aggregation to improve performance^65^. RF is an ensemble of unpruned decision tree models. It introduces randomness in the development of trees to achieve performance improvement. Firstly, RF uses distinct bootstrap sampling with replacement for each decision tree, enabling the selection of unique subsets of data. Secondly, during node splitting, RF randomly selects from a subset of features for each node in each tree, introducing randomness in the tree-building process. These random processes provide reduced correlation among trees.
- **Gradient Boosting Ensemble of Trees:** Gradient Boosting is a powerful machine-learning technique that builds predictive models in the form of an ensemble of weak prediction models (Decision trees). The basic idea is to train a series of models (weak learners) sequentially, with each subsequent model trained to correct the errors made by the previous one. LightGBM^66^ and XGBoost^67^ are two of the most advanced and efficient implementations of the gradient boosting framework, Both frameworks utilize an ensemble of decision trees to deliver powerful and accurate prediction models.

#### Probability estimation in ensemble of trees models for binary classification

An ensemble of trees model provides a way to return probabilities for classification by aggregating the votes of its decision trees and using the proportion of trees that vote for a specific class to estimate the probability of an instance belonging to that class. For example, to obtain probabilities for classification, the model uses the proportion of decision trees that vote for the positive class. For example, if there are 100 decision trees in the forest, and 70 of them vote for the positive class, the probability of the input belonging to the positive class would be 0.7. The final prediction from the model is obtained through the voting mechanism. Each decision tree “votes” for a particular class and the class with the most votes is considered the final prediction.

### Black box nature of the machine learning models

Machine learning models operate by accepting a set of features as input and generating outputs in the form of probability scores or class labels. Among our chosen models, logistic regression is the only transparent model, as we can discern the impact of individual feature variables on the model’s prediction.

The ensemble of trees is often considered a “black box” due to its complex and less interpretable internal workings compared to simpler models like logistic regression and singular decision tree. Comprising of multiple decision trees, the ensemble model’s combination enhances overall accuracy but increases complexity and opacity. The hierarchical structure of nodes and splits in each decision tree makes understanding the model’s decision-making process challenging, particularly as the number of trees and forest complexity grow. The model lacks explicit formulas or equations that describe the collective behaviour of the trees, contributing to the black box nature.

In contrast to Logistic Regression, both ANNs (Artificial Neural Networks) and SVMs (Support Vector Machines) construct hyperplanes within a transformed, non-linear space, making it difficult to directly assess how a specific feature value affects the model’s predictions.

Our notion of interpretability hinges on the ability to determine, for any given instance, how each feature impacts the model’s prediction. SHAP (SHapley Additive exPlanations) offers a model-agnostic approach to achieve precisely this objective.

## Model evaluation and SHAP-based interpretability

### Evaluation metrics

To ensure a standardized evaluation of our model and to provide more insights into our results, we calculate the Accuracy, Precision, Recall, F1 score, AUC under ROC and Mathews correlation coefficient (MCC) as the evaluation metrics.

The terms are defined as the following:

15 $$\begin{aligned} \text {Precision}&= \frac{\text {TP}}{\text {TP} + \text {FP}} \end{aligned}$$

16 $$\begin{aligned} \text {Recall} &= \frac{{\text {TP}}}{{\text {TP}} + {\text {FN}}} \end{aligned}$$

17 $$\begin{aligned} \text {F1 score} &= \frac{2 \times \text {Precision} \times \text {Recall}}{\text {Precision} + \text {Recall}} \end{aligned}$$

18 $$\begin{aligned} \text {MCC} &= \frac{{\text {TP}} \times {\text {TN}} - {\text {FP}} \times \text {FN}}{\sqrt{({\text {TP}} + {\text {FP}})({\text {TP}} + {\text {FN}})({\text {TN}} + {\text {FP}})({\text {TN}} + {\text {FN}})}} \end{aligned}$$

19 $$\begin{aligned} \text {Accuracy} &= \frac{{\text {TP}} + {\text {TN}}}{{\text {TP}} + {\text {TN}} + {\text {FP}} + {\text {FN}}} \end{aligned}$$

In these formulas, TP (True Positives) denotes the count of correctly identified positive cases, TN (True Negatives) represent the correctly identified negative cases, FP (False Positives) indicate the positive cases incorrectly classified, and FN (False Negatives) represent the negative cases that were incorrectly classified. These metrics provide essential insights into the performance of the model, with the F1 score balancing precision and recall, and the MCC offering a comprehensive measure of the model’s effectiveness across imbalanced datasets. The AUC, or Area Under the Curve of the Receiver Operating Characteristic (ROC) curve, serves as a singular metric to gauge the model’s ability to differentiate between classes across varying threshold settings, with the ROC curve plotting the True Positive Rate (TPR) against the False Positive Rate (FPR).

### Interpreting model-feature relationships with SHAP

Our primary goal with the feature engineering framework is to extract features that improve the distinction between AMPylated and non-AMPylated sites in a given protein sequence. To gain a comprehensive understanding of their usefulness, it is essential to grasp the impact of these extracted features on the model’s predictions. Hence, we employ an explanation technique called “SHAP” (Shapley Additive exPlanations) to shed light on the contribution of each feature towards the model’s decision-making process.

#### SHAP framework for black-box model explanations

SHAP (SHapley Additive exPlanations)^50^ is a model interpretability method based on Shapley values from cooperative game theory. It assigns each feature (cooperative player) an importance value for a particular prediction to explain the output of any machine learning model.

Shapley values offer an equitable approach for distributing the rewards or payouts among participants engaged in a cooperative game. In a cooperative game, there are *N* players who work together to produce a total payout of *v*. The Shapley value $\phi _i$ assigns a fair payout share to each player *i* while satisfying the following desirable properties:

1. Efficiency: The sum of payouts equals the total payout $\sum _{i=1}^N \phi _i = v$
2. Symmetry: Players contributing equally get equal payouts
3. Null player: Players not contributing get zero payout
4. Additivity: Payouts from separate games add up $\phi _i(v + w) = \phi _i(v) + \phi _i(w)$

Let’s consider a regression model *f* that takes an input ${x} \in \mathbb {R}^n$ and outputs a prediction *f*(*x*). Shapely values explain a prediction by computing the contribution $\phi _i$ of each feature $x_i$ to the output *f*(*x*).

The Shapley values $\phi _i$ are calculated according to the following equation:

20 $$\begin{aligned} \phi _{i} = \sum _{S \subseteq F\backslash { \{i\}}}^{} \frac{\vert S\vert !\left( \vert F\vert - \vert S\vert - 1\right) !}{\vert F\vert !}\left[ f\left( x_{S \cup { \{i\}}}\right) - f\left( x_{S}\right) \right] \end{aligned}$$

where *F* is the set of all features, and *S* is a subset of features that do not contain feature *i*.

This computes $\phi _i$ by considering all possible feature subsets *S* and evaluating how the prediction changes by including or excluding feature *i*. The change in *f* represents the impact of feature *i*.

The SHAP values explain the difference between the base value and the actual prediction. The higher the SHAP value magnitude $\vert \phi _i \vert$, the more important that feature is for that prediction.

In machine learning models, the input size is usually fixed and cannot handle inputs of varying sizes. When computing the Shapley values, $f(x_S)$ is replaced by the expected value $E[f(z)|x_S]$.

$E[f(z)|x_S]$ is the expected value of the model output *f*(*z*) conditioned on a subset of features *S* taking values from the input *x*. So it fixes the features in *S* to values from *x*, and marginalizes over the missing features by averaging over the training data distribution. By using expected values, SHAP extends the Shapley values computation to standard ML models.

The local accuracy property of SHAP values allows us to represent the output as:

21 $$\begin{aligned} f({x}) = \phi _0 + \sum _{i=1}^{n} \phi _i \end{aligned}$$

Where $\phi _i$ denotes the feature contribution of the ith feature which is also called its SHAP value, and the base value $\phi _0$ is *E*[*f*(*x*)].

SHAP is an invaluable tool for interpreting the probabilities returned by Random Forest in binary classification tasks. As an advanced interpretability technique, SHAP provides a comprehensive and intuitive understanding of the model’s decision-making process. By analyzing SHAP values, we can discern the relative importance of each feature in influencing the predicted probability for a particular data point. This helps identify the critical features driving the model’s decisions.

#### Scope of the explanations

Local explanations are centred on providing insights into individual predictions made by a model. Within this framework, SHAP generates additive feature contributions that reveal the impact of each feature on a specific prediction. These contributions aid in comprehending how each feature contributes to the outcome for a particular instance.

On the other hand, global explanations have the objective of understanding the overall behaviour of the model. SHAP takes into account an extensive set of instances sampled from the data distribution and generates a collection of local explanations, with each explanation corresponding to a specific instance. These local explanations consist of feature contribution values. To gain a broader understanding of the model’s behaviour beyond individual predictions, an analysis is conducted on the feature contribution values across all instances in the set. This analysis facilitates the understanding of how different features collectively contribute to the model’s predictions, providing valuable insights into the relationship between the model and the features as a whole.

## Experiments and results

To evaluate the effectiveness of our feature engineering framework, we employ k-fold cross-validation (k=10), dividing the dataset into k subsets. During each fold, k-1 subsets are utilized for training, while one subset is reserved for validation, ensuring that the entire dataset is utilized for testing the model. To maintain a consistent ratio of negative and positive sites in both training and validation datasets, we choose stratified k-fold cross-validation.

The feature engineering process involves specifying various parameter combinations to generate features. A selected model is then trained on the training set and evaluated on the test set in each fold by evaluation metrics outlined in the previous section. These evaluation metrics values are then aggregated over the k folds. No hyper-parameter tuning is performed and taken as the default values provided by the Python packages.

Through a comparative study, we sought to identify which features, based on the chosen parameters, engineered by our framework performed the best for the different choices of machine learning models. To facilitate this comparison, we create three lists of parameters: $L_1$ = [“NR”, “H”, “C”], $L_2$ = [“mono counts”, “bigram counts”, “texture features”, “pro2vec”], and $L_3$ = [1, 2, 3]. Using different combinations of subsets from $L_1$, $L_2$, and $L_3$, we train and evaluate the model, leveraging these parameters to create features. For each selected combination of subsets, we train and evaluate a separate model using the engineered features generated from those specific parameters. By using different subsets, we effectively explore a diverse range of features, allowing us to identify the most effective feature engineering setups for our evaluation.

The outcomes are presented in Table 1. However, due to space constraints, not all results are displayed. The Column “Nfeat” denotes the total count of engineered features. The table exhibits the feature sets that yield the best performance for each of the models sorted by F1-score.

**Table 1:** Detailed performance metrics of top performing models.

Model	Representations	Feat types	Offsets	Nfeat	Accuracy	Precision	Recall	F1 score	AUC ROC	MCC
ANN	(‘conform’, ‘no_reduction’)	(‘mat’,)	(1, 3)	898	0.804552	0.718347	0.757692	0.734459	0.853464	0.583993
XGB	(‘no_reduction’, ‘hydro’)	(‘counts’, ‘mat’)	(1, 3)	875	0.791181	0.698694	0.736264	0.714020	0.857740	0.553493
LGBM	(‘conform’, ‘no_reduction’)	(‘counts’, ‘mat’)	(1, 2, 3)	1374	0.799075	0.745368	0.691758	0.707679	0.859776	0.565576
RF	(‘conform’, ‘no_reduction’, ‘hydro’)	(‘counts’, ‘mat’)	(1, 3)	980	0.809744	0.829522	0.606044	0.687253	0.885388	0.580302
SVM	(‘no_reduction’,)	(‘counts’, ‘mat’)	(1, 2)	820	0.788834	0.741946	0.648352	0.686790	0.863235	0.536218
Linear	(‘no_reduction’, ‘hydro’)	(‘tfeat’, ‘pro2vec’)	(3,)	360	0.756757	0.655678	0.706044	0.672372	0.808359	0.488660

The ANN model achieves the highest F1-score at 0.73, utilizing bigram counts from two distinct representations with offsets 1 and 3, totalling 898 features. Meanwhile, the Random Forest model stands out with the highest AUC-ROC score, reaching 0.88, alongside an MCC of 0.5803 and an F1 score of 0.6873, across 10 folds. This performance is achieved through a comprehensive feature set derived from all three representations, incorporating monogram and bigram counts for offsets 1 and 3, totalling 980 features. Interestingly, the logistic regression model records an F1 score of 0.67 without relying on counts but instead leveraging texture features and Pro2Vec embeddings. These results demonstrate the potential benefit of considering diverse feature combinations and representations to enhance the model’s overall performance.

**Table 2:** Detailed performance metrics of top performing models with less than 100 features.

Model	Representations	Feat types	Offsets	Nfeat	Accuracy	Precision	Recall	F1 score	AUC ROC	MCC
XGB	(‘conform’, ‘no_reduction’)	(‘counts’,)	(1,)	27	0.772617	0.675893	0.698352	0.683290	0.816823	0.509881
RF	(‘no_reduction’,)	(‘counts’, ‘tfeat’, ‘pro2vec’)	(1,)	75	0.791110	0.746792	0.639560	0.681438	0.851257	0.537578
LGBM	(‘conform’, ‘no_reduction’)	(‘counts’,)	(1,)	27	0.767354	0.674866	0.698352	0.675327	0.824072	0.506217
ANN	(‘no_reduction’, ‘hydro’)	(‘counts’, ‘tfeat’)	(3,)	35	0.727383	0.612973	0.773077	0.669234	0.787573	0.475561
Linear	(‘no_reduction’,)	(‘tfeat’, ‘pro2vec’)	(1, 2, 3)	65	0.732788	0.646500	0.613187	0.619096	0.785228	0.424621
SVM	(‘no_reduction’,)	(‘counts’,)	(1,)	20	0.719772	0.637944	0.559890	0.587702	0.805160	0.386226

Having a compact yet effective feature set is crucial, which is why the results for models with fewer than 100 features are also presented, ranked by F1 score across 10-fold cross-validation. These findings are detailed in Table 2.

Given the small size of our dataset, we opted for 10-fold cross-validation to ensure robustness. Additionally, we partitioned the data using an 80:20 split for training and testing, respectively. We then conducted experiments using the models and features outlined in Table 1, with the outcomes presented in Table 3. The models are trained on the training data and evaluated over the test data.

**Table 3:** Detailed performance metrics of various models on the test split of the dataset.

Model	Representations	Feat types	Offsets	Nfeat	Accuracy	Precision	Recall	F1 score	AUC ROC	MCC
ANN	(‘conform’, ‘no_reduction’)	(‘mat’)	(1, 3)	898	0.815789	0.724138	0.777778	0.750000	0.900605	0.605428
SVM	(‘no_reduction’)	(‘counts’, ‘mat’)	(1, 2)	820	0.802632	0.772727	0.629630	0.693878	0.913076	0.556759
XGB	(‘hydro’, ‘no_reduction’)	(‘counts’, ‘mat’)	(1, 3)	875	0.776316	0.678571	0.703704	0.690909	0.867725	0.515952
LGBM	(‘conform’, ‘no_reduction’)	(‘mat’)	(1, 2, 3)	1347	0.750000	0.642857	0.666667	0.654545	0.879819	0.458957
RF	(‘conform’, ‘no_reduction’, ‘hydro’)	(‘counts’, ‘mat’)	(1, 3)	980	0.789474	0.823529	0.518519	0.636364	0.908541	0.525200

### SHAP analysis of model-features combination

As previously mentioned, many of the models under review operate as black boxes, obscuring their internal decision-making processes. To render the predictions of these models interpretable, SHAP (SHapley Additive exPlanations) can be utilized, attributing the output of any model to the contributions of each feature involved. For our SHAP analysis, we have chosen the Random Forest model, which utilizes 980 features, including both monogram and bigram counts across all sequence representations. This selection is also motivated by the fact that breaking down predictions into the effects of different counts makes the model interpretable in a meaningful way.

**Figure 3:**
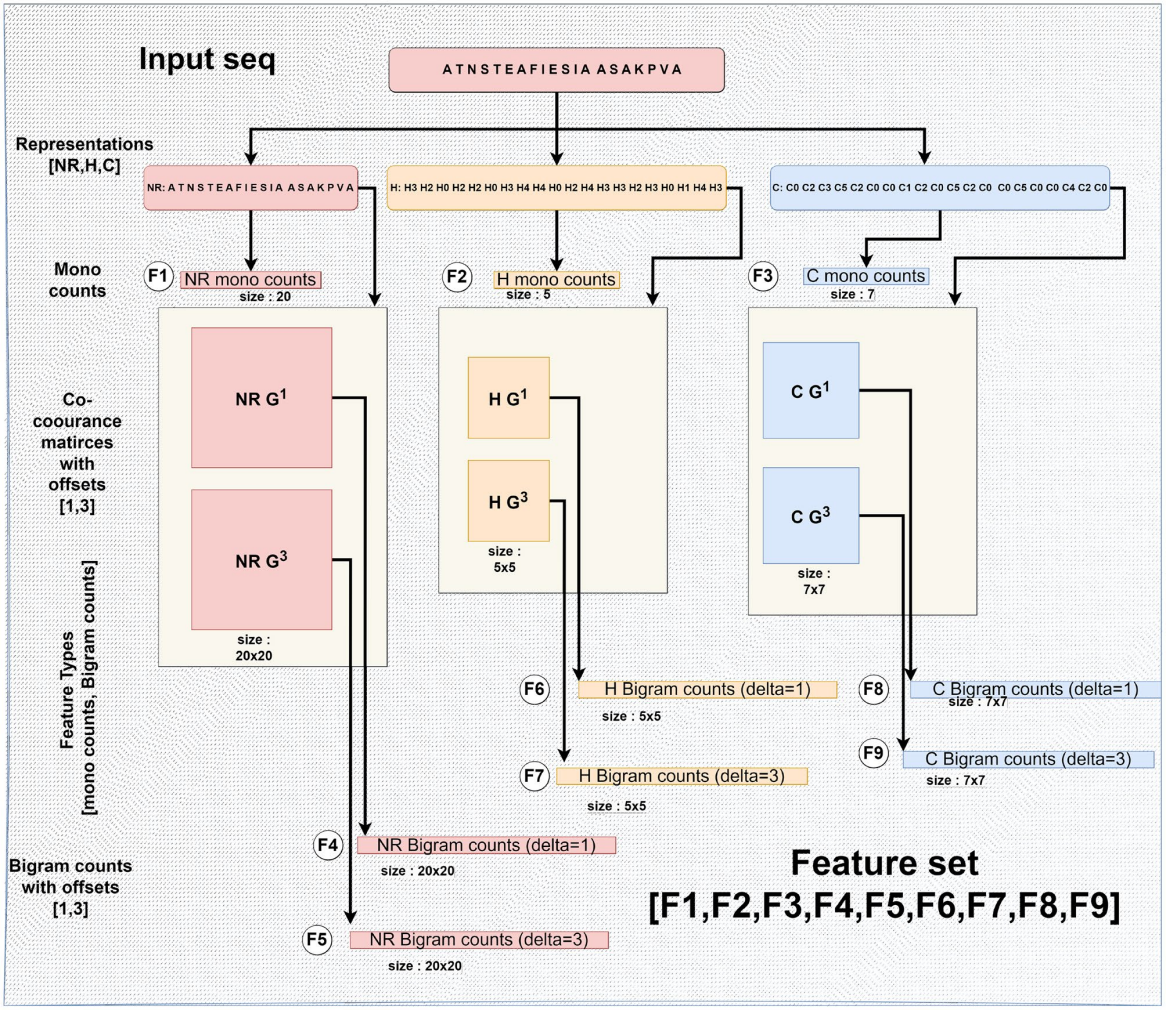
980 features extracted by using the framework. Each of the representations is processed separately, and the count vectors generated are concatenated to create the final set of features.

The optimal model, achieving an average AUC-ROC of 0.88, MCC of 0.58 and an F1 score of 0.69 over 10 folds, is attained by utilizing all three representations and generating features from monogram counts. The bigram counts for offsets 1 and 3. This feature set consists of a total of 980 features.

First, we select all three sequence representations—S1 (NR), S2 (H), and S3 (C). These sequences are processed independently. Next, we select the feature types—monogram counts and $\delta$-bigram counts derived from the co-occurrence matrix. Sequences S1, S2, and S3 are processed to obtain monogram count vectors of sizes 20, 5, and 7 respectively. The sequence S1 may consist of 20 distinct characters, S2 of 5, and S3 of 7. The normalized frequency of each character’s occurrence in the sequence corresponds to the value at the position of that character in the vector of monogram counts. Similarly, co-occurrence matrices are computed with bi-gram combinations of characters. We also derive 20 × 20, 5 × 5, and 7 × 7 bigram counts from the co-occurrence matrices for S1, S2, and S3. We use offsets of 1 and 3, so two co-occurrence matrices are created for each sequence and offset. This results in four matrices per sequence from which bigram counts are extracted. Finally, all the different features are concatenated into a single vector. With our selected parameters, we get: $5 + 20 + 7$ (monogram counts), $20\times 20 + 5\times 5 +7\times 7$ ($\delta =1$ bigram counts), and $20\times 20 + 5\times 5 +7\times 7$ ($\delta =3$ bigram counts), totaling 980 features. The procedure is shown in terms of a flowchart in Fig. 3.

### SHAP explanations

Our objective is to further explore the connection between the model and the high-performing feature set generated through our feature engineering framework (nfeat = 980). To accomplish this, we divide the data into an 80:20 ratio, maintaining an equal distribution of classes in both segments. The training protein data is different from the test protein data. Following this, we train the model on the training subset and evaluate its effectiveness on the test subset. Once we validate its performance on the test set, we employ SHAP analysis to gain a deeper understanding of how our black-box model makes decisions, thereby enhancing its interpretability. The model’s performance on the test split is quantified by two key metrics. The F1 score achieved is 0.6364. Additionally, the Matthews correlation coefficient (MCC) is 0.5252, which suggests a reasonable quality of binary classifications made by the model. As we move forward, let’s establish key terms. monogram counts are denoted by their respective characters, while bigram counts are indicated as “AB($\delta$)”, where A and B are characters and $\delta$ signifies the offset in co-occurrence matrix construction.

**Figure 4:**
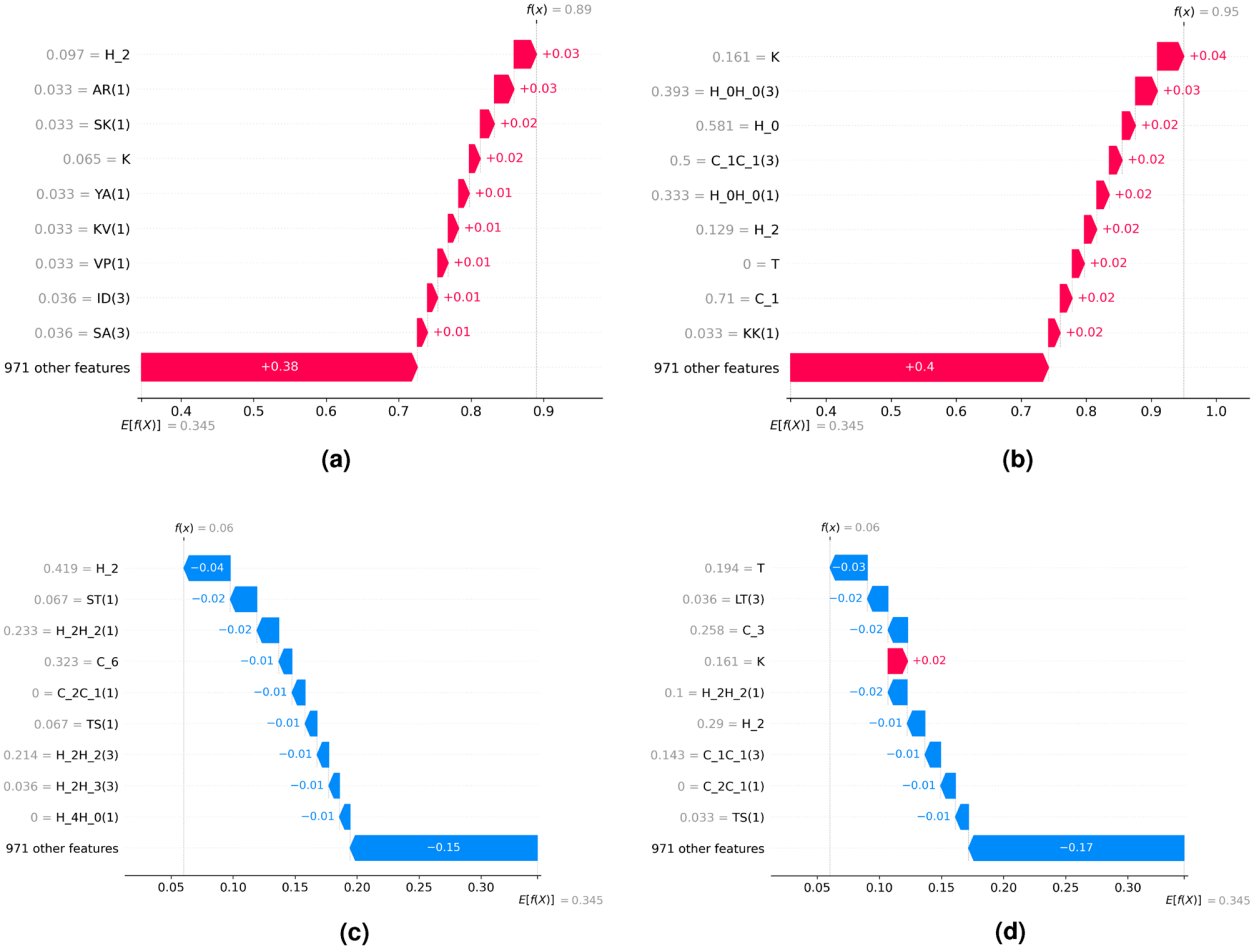
Local explanations using waterfall plots. A prediction *f*(*x*), for instance, *x*, and its deviation from *E*[*f*(*x*)] could be measured as the sum of the feature importance values (SHAP values) of individual features. (**a**) Instance with positive prediction (p = 0.89) (**b**) Instance with positive prediction (p = 0.95) (**c**) Instance with negative prediction (p = 0.06) (**d**) Instance with negative prediction (p = 0.06).

**Figure 5:**
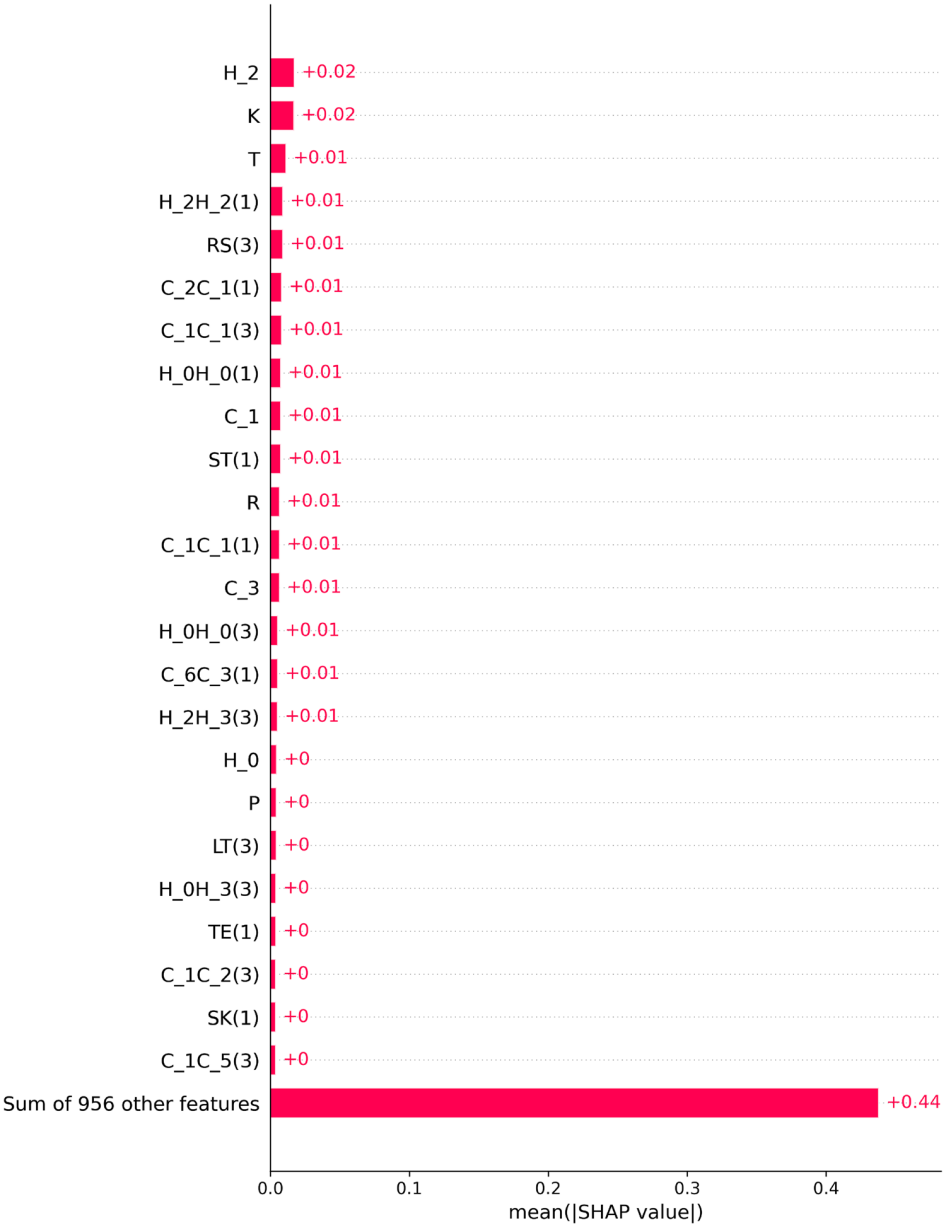
Global feature importance. It is the expected composition of feature importance score (SHAP values) estimated by aggregating of SHAP values across various predictions coming from the data distribution.

**Figure 6:**
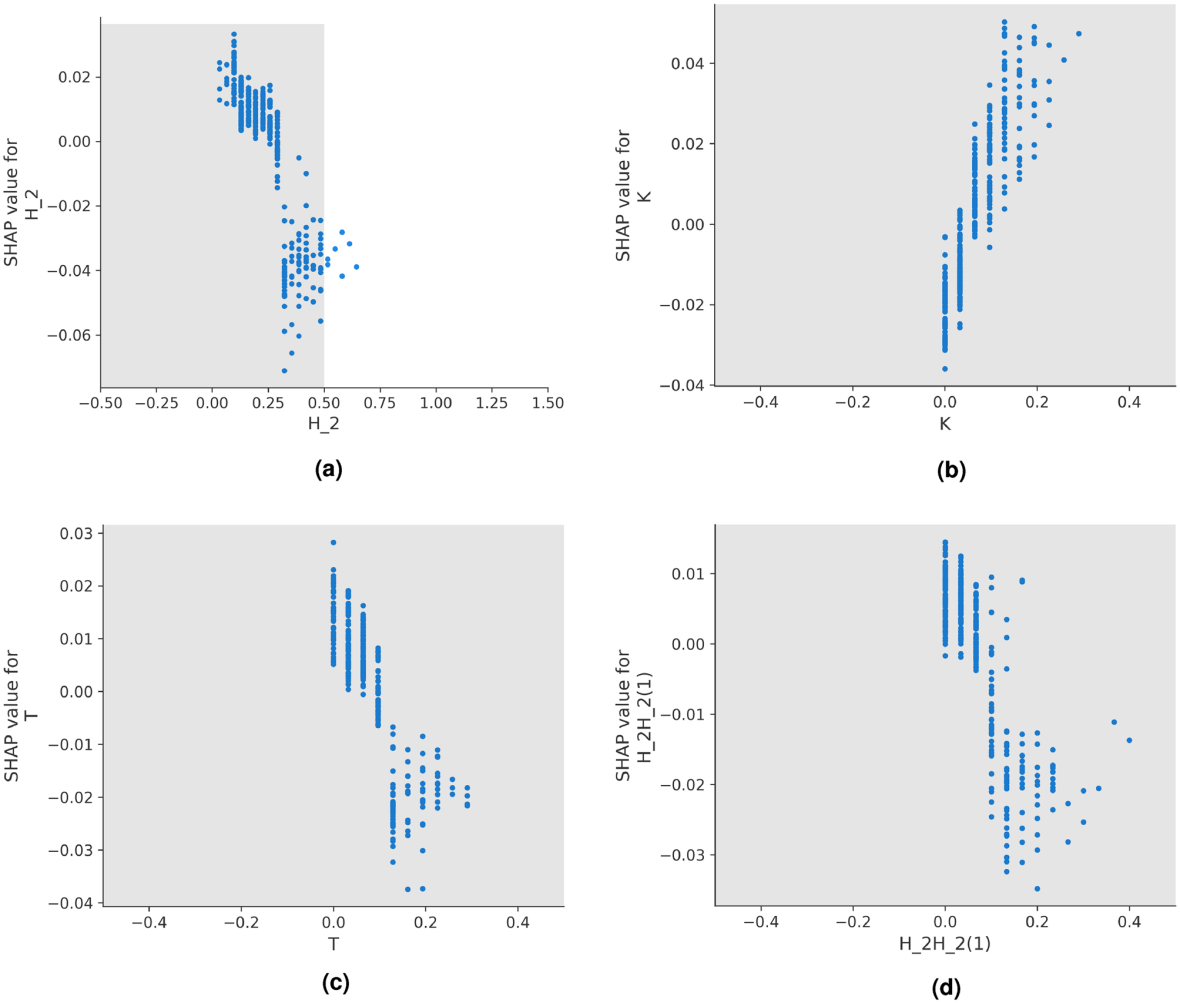
SHAP scatter plot for capturing the trend in the importance value. (**a**) SHAP scatter plot for feature H_2 (**b**) SHAP scatter plot for feature K (**c**) SHAP scatter plot for feature T (**d**) SHAP scatter plot for feature H_2H_2(1).

To explain the model’s behaviour locally for a specific instance, we calculate SHAP values and present them in the form of a waterfall plot. This plot illustrates the individual contributions of each feature to the model’s prediction output. By analyzing the local explanations, users can gain valuable insights into the reasons behind the model’s specific predictions for given inputs. The waterfall plot comprises horizontal bars that represent different features, stacked vertically (in order of impact). The length of each bar reflects the impact of the corresponding feature on the prediction. Figure 4 displays waterfall plots for four distinct instances from the test dataset. The base prediction value, which represents the average prediction over the training set for the model, is 0.345. The upper subfigures, referenced as Fig. 6a,b, depict two instances for which the model exhibits strong confidence in assigning them to the positive class. Conversely, the lower subfigures, labelled Fig. 6c,d, represent two instances that the model confidently categorizes into the negative class. Across all subfigures, the influence of curated features like “H_2” is evident. These examples highlight that as the quantity of amino acids within the “H_2” group decreases, the effect on yielding a positive prediction also diminishes.

The global-level explanations shed light on the general importance of features to the black-box model, emphasizing their overall significance. Additionally, these global explanations offer valuable insights into the trends and patterns in feature importance across various instances. However, conducting this analysis necessitates a sufficiently large and representative dataset that aligns with the data distribution. To perform the global-level analysis, a SHAP coefficient matrix is generated. This process involves making SHAP calls on all instances in the dataset and storing the resulting coefficients for each feature in a matrix. The matrix has the same number of columns as features and rows as instances, facilitating a comprehensive examination of feature contributions on a broader scale.

To assess the extent of feature significance across all instances, we calculate the average absolute SHAP values for each feature. This involves computing the mean of the absolute values within each column of the SHAP coefficient matrix. In Fig. 5, a bar plot is presented to visualize the global feature importance. This plot illustrates the overall impact of a specific feature on the model’s predictive process.

While analyzing the specific explanations for the instances illustrated in Fig. 4, we identified a notable pattern in the SHAP values associated with the “H_2” feature. To determine whether this pattern is consistent across a broader spectrum and to uncover trends within the top 4 globally significant features according to SHAP, we employ the SHAP matrix to construct scatter plots. These scatter plots provide a visual representation of the connection between SHAP values along the y-axis and the corresponding feature values of instances (across the entire dataset) along the x-axis. The scatter plots are shown in Fig. 6. The features “H_2”, “T” and “H_2H_2(1)” have a downward trend, while the feature “K” has a positive trend. These features correspond to the counts of the corresponding amino acid characters. The feature “H_2H_2(1)” indicates the co-occurrence of two amino acids belonging to the group “H_2” ($\delta =1$).

## Discussion

In this work, we have introduced a comprehensive feature engineering framework for extracting informative representations from protein sequences to improve AMPylation site prediction. Our framework allows combining multiple sequence representations, feature types, and offsets to generate diverse and discriminative features.

Through systematic experiments using cross-validation, we identified an optimal combination of sequence representations, feature types, and offsets that yielded good results for all the different models selected with no hyperparameter tuning. This demonstrates the capability of our framework to engineer an effective set of features for discerning between AMPylated and non-AMPylated sites. The top-performing set of features extracted achieved MCC score of 0.58, Accuracy of 0.8, AUC-ROC of 0.85 and F1 score of 0.73.

Despite the availability of previous work done by Azim et al.^20^ on GitHub, which includes the full dataset and a trained model file, the lack of detailed training configurations, the model’s constructor class definition, and explicit information on their train-test split prevents us from creating a new model or using the existing model file without risking train-test data overlap. Nevertheless, in our revised study, we have expanded our analysis to include a variety of models. It’s noteworthy to mention that their research achieved a 10-fold cross-validation performance with 77.7% accuracy, 79.1% sensitivity, 76.8% specificity, a Matthews Correlation Coefficient of 0.55, and an Area Under Curve of 0.85. These results are on par with our simpler models, which do not involve hyper-parameter tuning, though our train-test splits during the 10-folds may vary.

Addressing the need for explainability in machine learning, particularly for the inherently black-box Random Forest model, we utilized SHAP values to furnish both local and global explanations. Local explanations provided clarity on the impact of specific features (monogram and bigram counts) on individual predictions, while global insights shed light on the relative importance of features overall.

In conclusion, our proposed feature engineering framework enables building a high-performing data-driven approach for the crucial task of AMPylation site prediction. The framework provides flexibility to generate and evaluate diverse features. In future work, our approach could be extended to other PTM prediction tasks and other popular binary classifiers. Overall, this study demonstrates the significant potential of tailored feature engineering and model explainability techniques for advancing computational prediction of post-translational modifications.

## Data Availability

The codebase including the dataset used is present at https://github.com/HardikPrabhu/Protein-Feature-Engineering-Framework-for-AMPylation-Site-Prediction. The data that support the findings of this study is originally available at https://github.com/MehediAzim/DeepAmp.
